# PREVALENCE AND FACTORS ASSOCIATED WITH MOTORIC COGNITIVE RISK IN A COMMUNITY-DWELLING OLDER SCOTTISH POPULATION

**DOI:** 10.1093/geroni/igac059.2270

**Published:** 2022-12-20

**Authors:** Donncha Mullin, Lucy Stirland, Tom Russ, Michelle Luciano, Graciela Muniz Terrera

**Affiliations:** University of Edinburgh, Edinburgh, Scotland, United Kingdom; University of Edinburgh, Edinburgh, Scotland, United Kingdom; University of Edinburgh, Edinburgh, Scotland, United Kingdom; University of Edinburgh, Edinburgh, Scotland, United Kingdom; Ohio University, Athens, Ohio, United States

## Abstract

Motoric Cognitive Risk (MCR) syndrome combines slow walking and self-reported cognitive complaints. It is a quick and simple way of identifying individuals at high risk of developing dementia. MCR has not been described in a Scottish population to date. This study describes the prevalence and associated factors of MCR in a community-dwelling sample of older Scottish people. The MCR concept was derived in the Lothian Birth Cohort 1936 (LBC1936) - a highly phenotyped cohort of over 1000 people followed up every 3 years since 2004. Uniquely, participants in LBC1936 had their IQ measured at age 11 in 1936. Authors found MCR prevalence of approximately 5.4% at baseline. Using logistic and linear regression analysis, as appropriate, they found that participants' age, depressive symptoms and cognitive measures of executive function were significantly associated with an increased likelihood of have MCR, but that IQ aged 11 was not associated. This study found rates of MCR in Scotland are within the typical range for this age group, albeit on the lower end. Interestingly, IQ at age 11 was not significantly associated with MCR, which was unexpected given MCR's prognostic value for dementia. That tests of executive function were associated with MCR adds further credence to the hypothesis that walking speed and executive function are linked. This points to further important work to ascertain if increasing walking speed can improve executive function.

